# Construction of a series of vectors for high throughput cloning and expression screening of membrane proteins from *Mycobacterium tuberculosis*

**DOI:** 10.1186/1472-6750-8-51

**Published:** 2008-05-16

**Authors:** Huajun Qin, Jian Hu, Yuanzhi Hua, Shridhar V Challa, Timothy A Cross, Fei P Gao

**Affiliations:** 1 Department of Chemistry and Biochemistry, Florida State University, Tallahassee, Florida 32310, USA; 2 National High Magnetic Field Laboratory, Tallahassee, Florida 32306, USA; 3 Scripps Research Institute, La Jolla, California 92037, USA; 4 Institute of Molecular Biophysics, Florida State University, Tallahassee, Florida 32306, USA

## Abstract

**Background:**

One of the major challenges for membrane protein structural genomics is establishing high-throughput cloning and expression screening methods to obtain enough purified protein in a homogeneous preparation for structural and functional studies. Here a series of ligation independent cloning based vectors were constructed to address this challenge.

**Results:**

The feasibility of these vectors was tested with 41 putative membrane proteins from *Mycobacterium tuberculosis*. The efficiency for direct cloning of these target genes from PCR products was 95% (39/41). Over 40% of cloned genes were overexpressed in *Escherichia coli *BL21 (DE3)-RP codon plus strain in the first round of expression screening. For those proteins which showed no expression, three protein fusion partners were prepared and it was found that each of the target proteins could be overexpressed by at least one of these fusions, resulting in the overexpression of two thirds of the cloned genes.

**Conclusion:**

This expression platform features high throughput cloning, high flexibility for different constructs, and high efficiency for membrane protein overexpression, and is expected to be useful in membrane protein structural and functional studies.

## Background

Genomic sequence analysis predicts that integral membrane proteins constitute 20–30% of all sequenced prokaryotic and eukaryotic genomes. These proteins are critical for many essential cellular functions and constitute 60 to 70% of current drug targets [1]. However, to date, less than 1% of the atomic resolution structures in the Protein Data Bank represent membrane proteins [2]. The remarkable gap between the significance of membrane proteins and the limited number of high resolution membrane protein structures is, in part, due to the availability of membrane proteins for structural biology.

For structure determination, obtaining enough highly homogeneous protein is the first substantial challenge, especially for membrane proteins [3-5]. For a specific membrane protein, overexpression of a functional state is so difficult that researchers often have to perform numerous expression trials. Apart from selection of a suitable strain as the host and optimization of expression conditions, vector construct optimization can be critical, including selection of promoters, fusion partners, truncation, mutation and other protein engineering methods [6-10]. Such "trial and error" processes can be very time consuming.

The ligation independent cloning (LIC) approach was developed for direct cloning of PCR products without restriction enzyme digestion or ligation reactions [11,12]. By making use of the 3'–5' exonuclease activity of T4 DNA polymerase, the specific 12–15 nucleotide single stranded overhangs can be created for both the vector and the insert. After annealing *in vitro *and transformation into the host cells, the vector and the insert are covalently ligated *in vivo *to form a circular plasmid. Generally, the LIC approach features simplicity and very low non-recombinant background, and it is suitable for high throughput cloning [13-15].

There is no doubt that the construction of a high throughput cloning and expression screening platform would facilitate expression optimization through construction of fusion proteins and protein engineering as well as the characterization of membrane proteins. In the present effort, we have developed such a platform, which consists of a series of ligation independent clone (LIC) based vectors, for high throughput cloning and expression screening and then tested the platform with 41 putative integral membrane proteins from *Mycobacterium tuberculosis*. These targets have a molecular weight range lower than 30 kDa and one to four transmembrane α-helices. The results show that, by using this platform, genes encoding membrane proteins can be efficiently cloned for initial expression screening. For those proteins which could not be well expressed in the initial screening, three fusion partners were used to successfully facilitate their overexpression.

## Methods

### Construction of expression vectors

**Figure 1:**
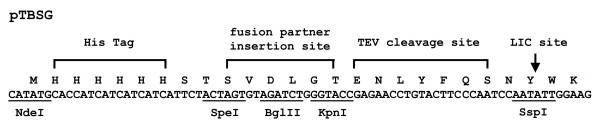
Sequence and illustration of pTBSG and its derivatives. Following the His tag at the very N terminus, different fusion partners (KSI, GST and MSP) were inserted between the restriction enzyme sites for SpeI and KpnI. A tobacco etch virus protease (TEV) cleavage site was inserted between the fusion partner protein and the target protein which was inserted at the SspI site through LIC.

In the present work, the vectors used were modified from the LIC expression vector pMCGS7 [13] (a generous gift from Dr. Mark I. Donnell from University of Wisconsin). To facilitate the addition of fusion partner proteins onto the N terminus, a SpeI restriction site was introduced into the pMCSG7 vector right after the His tag by PCR (sense primer 5'-CATC ATTCTACTAGTGTAGATCTG-3', anti-sense primer 5'-CAGATCTACACTAGTAG AATGATG-3'), resulting in a new vector, named pTBSG. The maltose binding protein (MBP) gene was amplified by PCR by using the sense primer 5'-GGACTAGTAAAATCGAAGAAGGTAAACTG-3' and anti-sense primer 5'-CGGG GTACCAGTCTGCGCGTCTTTCAG-3'. The glutathione S-transferase (GST) gene was amplified by using the sense primer 5'-GGACTAGTCTAGGTTATTGGA AAATTAAG-3' and the anti-sense primer 5'-CGGGGTACCATCCGATTTT GGAGGATGGTC-3'. The ketosteroid isomerase (KSI) gene was amplified by using the sense primer 5'-GGACTAGTCATACCCCAGAACACATC-3' and the anti-sense primer 5'-GGGGTACCCTGGCATGCGTGAATATTC-3'. The PCR products were digested with restriction enzymes SpeI and KpnI, and then ligated into the pTBSG vector digested with the same enzymes, resulting in 4 fusion vectors (Figure 1).

### Cloning

Methods for target selection of membrane proteins from *Mycobacterium tuberculosis *were used as described [2]. The molecular weight of the target proteins were limited to about 30 kDa (Table 1). Target genes were amplified by PCR using a pair of primers in which the sense primer began with the sequence 5'-TACTTCCAATCCAATGCA-3' followed by the target gene and the anti-sense primer began with the sequence 5'-TTATCCACTTCCAATG-3' followed by the complement of a stop codon and the C-terminus of the target gene. The volume of a typical reaction mixture was 25 μL, the product was cleaned using a YM-30 spin column (Microcon, Inc.) and recovered in 50 μL of buffer including 50 mM Tris (pH 8.0) and 1 mM EDTA. The vector (with or without fusion partners) was digested with Ssp1 for two hours and applied to DNA agarose electrophoresis. The band corresponding to the cleaved vector was carefully sliced and recovered from the gel using the QIA^® ^gel extraction Kit (Qiagen) and then treated with T4 DNA polymerase (Novagen, LIC quality) in the presence of dGTP. The insert was treated with dCTP and T4 DNA polymerase at room temperature for 30 minutes then heated at 75°C for 20 minutes to stop the reaction. Annealing was carried out simply by mixing 1 μL of the digested vector, 2 μL of the insert and 1 μL of EDTA (25 mM, pH 8.0) and incubated at room temperature for 5 minutes. The annealed plasmid was transformed into DH5α competent cells. Positive clones were screened by PCR and then sequenced. Cloned genes were transformed into the expression host, BL21(DE3)-RP codon plus (Stratagene).

**Table 1:** Cloning and first round of expression screening of the targeted membrane proteins.

ORF	MW (kDa)	# of TM	Cloned	Expression location *	Expression Detection**	Expression Level ***
Rv0007	31	2	Yes	IB/M	w/w	++
Rv0008c	15.7	1	Yes	IB/M	c/c	+++
Rv0010c	15.2	2	Yes	IB/M	c/w	+++
Rv0011c	10.2	2	Yes	No		
Rv0012	28.3	1	Yes	IB/M	c/w	++++
Rv0150c	9.6	2	Yes	IB	c	++++
Rv0258c	16	2	Yes	IB/M	c/c	++++
Rv0345	14	2	Yes	IB	c	++++
Rv0420c	15.1	2	Yes	IB	c	++++
Rv0424c	10	1	Yes	IB/M	c/c	++++
Rv0426c	13.9	2	No			
Rv0460	8.1	3	Yes	IB	w	++
Rv0513	19.4	2	Yes	IB/M	w/w	+
Rv0514	10.3	2	Yes	IB	w	+
Rv0531	11.4	2	Yes	IB/M	w/w	++
Rv0544c	9.7	2	Yes	No		
Rv0882	9.6	3	Yes	No		
Rv1171	15.2	4	Yes	IB/M	w/w	++
Rv1214c	10.8	1	Yes	IB	c	++++
Rv1303	16.9	4	Yes	IB/M	c/c	+++
Rv1382	18.2	2	Yes	IB/M	w/w	++
Rv1567c	10.4	2	Yes	IB/M	c/c	+++
Rv1761c	13.5	1	Yes	IB/M	c/c	++++
Rv1772	10.9	2	Yes	No		
Rv1811	24.8	4	Yes	M	w	+
Rv2044c	11.9	3	Yes	IB/M	w/w	++
Rv2076c	9	2	Yes	IB/M	w/w	++
Rv2081c	14.2	1	Yes	IB/M	c/c	+++
Rv2128	7.4	2	Yes	No		
Rv2390c	19.9	1	Yes	IB/M	w/w	++
Rv2551c	13.8	4	Yes	No		
Rv2654c	7.7	2	No			
Rv2668	18.3	2	Yes	IB	c	+++
Rv2828c	19.6	2	Yes	IB/M	c/c	++++
Rv2843	17.7	3	Yes	IB	c	++++
Rv3078	14.1	4	Yes	No		
Rv3346c	8.9	2	Yes	No		
Rv3486	16	3	Yes	IB/M	w/w	++
Rv3632	13.1	3	Yes	No		
Rv3656c	7.1	1	Yes	No		
Rv3901c	15.4	1	Yes	IB/M	w/w	++

* IB: Inclusion body fraction; M: Membrane fraction.

** Protein expression was detected by Coomassie blue stain (c) or Western blot (w) after SDS PAGE.

*** Estimated expressed level: + < 0.5 mg/L; ++: 0.5–2 mg/L; +++: 2–10 mg/L; ++++: > 10 mg/L.

### Protein expression screening

The *E. coli *cells harboring the expression vector were grown on LB agar plates containing 50 μg/mL ampicillin and 34 μg/mL chloramphenicol. A single clone was picked and inoculated into 3 mL LB media for overnight growth. 100 μL of the overnight culture was then inoculated into 10 mL LB media, and the expression was induced with the addition of 0.4 mM IPTG when OD_600 _reached 0.6. The culture was grown for an additional 4 hours at 37°C. Cells were harvested by centrifugation at 4500 *g *for 15 min at 4°C, resuspended in 1 mL lysis buffer (10 mM Tris-HCl, pH 7.8, 5 mM EDTA) and lysed by sonication (Sonic Dismembrator, Model 100, Fischer Scientific, Inc.) three times (15 sec each). The lysate was fractionated by centrifugation for 20 min at 10,000 *g*. The supernatant normally contained soluble proteins and fragmented membranes, while the pellet consisted of insoluble proteins (inclusion body fraction). The supernatant was subjected to ultracentrifugation at 100,000 *g *for 45 min at 8°C to separate the membrane and soluble protein fractions. The soluble, insoluble and membrane fractions were adjusted to the same volume with lysis buffer, and then 15 μL of each was mixed with 5 μL sample buffer (4×) and 10 μL was loaded on 12% Tricine SDS-PAGE gels followed by either Coomassie staining or western blot using antibody against the His-tag of expressed proteins. Control experiments were performed under the same experimental conditions without IPTG induction.

### Small scale purification of Rv0011c

MBP-Rv0011c was expressed in 10 mL cultures as described above. The membrane fraction was collected and solubilized in 20 mM Tris-HCl, pH 7.8, 400 mM NaCl, and 1% DPC at 4°C for 1 hour. After ultracentrifugation at 100,000 *g *for 45 min at 8°C, the supernatant was mixed with 100 μL Ni^2+^-NTA resin (Qiagen) which was pre-equilibrated with the wash buffer (20 mM Tris-HCL, pH 7.8, 400 mM NaCl, 0.2% DPC and 5 mM imidazole). After incubation at 4°C overnight with gentle shaking, the resin was extensively washed with the wash buffer and then eluted with the elution buffer (20 mM Tris-HCl, pH 7.8, 400 mM NaCl, 300 mM imidazole and 0.2% DPC). TEV with an N terminal His tag was purified as reported previously [8] and added at a mass ratio of 5:1 (MBP-Rv0011c: TEV). The cleavage reaction was performed at 30°C for 2 hours. Before re-loading on the Ni^2+^-NTA resin (approximate 4 mg of fusion protein/mL resin) to remove MBP and TEV (both have an N terminal His tag), the sample was dialyzed against the dialysis buffer (20 mM Tris-HCl, 400 mM NaCl, 0.2% DPC, pH 7.9) for 4 hours to remove imidazole. The re-loading was performed at 4°C overnight with gentle shaking, and then the flow through containing the released Rv0011c was collected and analyzed by SDS-PAGE.

## Results and discussion

### Cloning through LIC

As shown in Table 1, 41 open reading frames (ORFs) identified in the *M. tuberculosis *genome as putative helical membrane proteins were targeted. These proteins range in molecular weight from 7.4 to 31 kDa with 1 to 4 putative transmembrane helices, similar to the target genes studied in a previous effort [2]. Only two of these targeted genes failed to be cloned using our LIC method, while the conventional ligase dependent method used previously resulted in a cloning success rate of only 72% [2]. This substantial increase in cloning efficiency can be attributed to the simplicity of ligation independent cloning. The most difficult step in the ligase dependent method is ligation, whose efficiency is strongly influenced by the sequence of the sticky end, the efficiency of the restriction endonuclease enzyme digestion and the experimental conditions. For LIC the ligation is performed *in vivo *by the intracellular system in the host cell, thereby avoiding the *in vitro *difficulties and increasing the cloning efficiency. LIC has been successfully used in high throughput cloning for soluble proteins [16], and the cloning efficiency in the present work is close to that for soluble proteins, indicating that this LIC method is also effective for high throughput cloning of membrane proteins.

### First round expression screening

**Figure 2:**
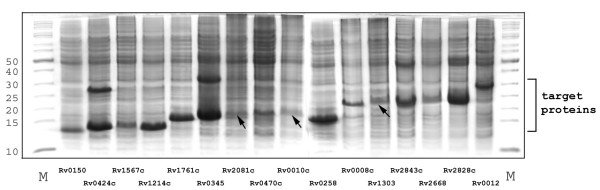
Overexpression of 16 putative membrane proteins in the first round of expression screening. After induction at 37°C for 4 hours, cells were harvested and lysed by sonication. The whole cell samples were subjected to SDS-PAGE. The gels were stained by Coomassie blue and the expressions were confirmed by western blot using an antibody against the His-tag. The arrows indicate the expressed proteins for lanes where it is not obvious.

Table 1 summarizes the results of the first round expression screening. Of the 39 cloned genes, 30 of them could be expressed as detected by Coomassie blue staining or western blot. Figure 2 shows the overexpression of 40% of these successfully cloned targets (16 proteins) detected by Coomassie blue staining. Although the expression level varied among these proteins, the yields are all estimated to be higher than 2 mg/L based on our experience with the Coomassie blue staining.

As in the previous study [2], it was found that many overexpressed membrane proteins were in the inclusion body fraction. The advantage of such expression is that the protein is often produced in large quantity (sometimes half of the whole cell protein mass) and in relatively pure form. Proteins expressed in inclusion bodies are also protected from proteolysis. Despite these advantages there are concerns about the refolding of proteins from inclusion bodies. However, methods have been designed to recover correctly folded proteins from these amorphous aggregates [17] and recently there have been numerous examples of successful refolding [18-21], including α helical membrane proteins [22-24]. The work from Villaverde's group showed two important features of these precipitated aggregates, namely that they are structurally and dynamically heterogeneous, but importantly, they provided evidence that the aggregation in the inclusion bodies is reversible [25], consistent with the recent refolding efforts. 21 of the membrane protein targets here, are also detected in the membrane fraction, and 8 proteins, including Rv0008c, Rv0258c, Rv0424c, Rv1303, Rv1567c, Rv1761c, Rv2081c and Rv2828c, were found to be overexpressed (≥2 mg/L) in the membrane fraction. Expression in the membrane fraction is a strong indication that the protein may be in its native or native-like conformation and that the *in vitro *refolding process can be avoided in sample preparations for various characterizations.

### Second round expression screening as fusion proteins

**Figure 3:**
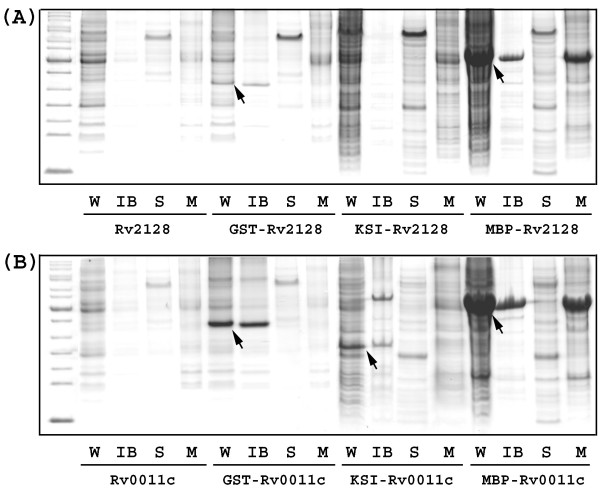
Expression of Rv2128 (A) and Rv0011c (B) in different constructs. Cells were harvested and then sonicated (W: whole cell). After centrifugation at 10,000 *g *for 20 min, the inclusion body fraction (IB) was collected. The supernatant was ultracentrifuged at 100,000 *g *for 45 min, and the supernatant (soluble fraction (S)) and pellet (membrane fraction (M)) were collected, respectively. The arrows indicate the overexpressed fusion proteins. Note that Rv2128 and Rv0011c could not be overexpressed without fusion partners, and there was no detectable expression for the KSI-Rv2128 fusion.

To improve upon the first round efforts, three commonly used fusion partners (MBP [26], GST [27] and KSI [28]) were tested for the 10 putative membrane proteins that did not express in the first round of screening. Table 2 lists the results for cloning and expression screening of the fusion proteins where expression was detected by Coomassie blue staining. Since the same LIC site was used in all the vectors (Figure 1), T4 DNA polymerase treated PCR products in the first round of screening were inserted directly into these vectors without any additional treatment. All the target genes were successfully inserted into the fusion protein vectors and expressed at high level from at least one of the fusion vectors. Figure 3 shows the gels for expression of Rv 0011c and Rv 2128 from the various vectors.

**Table 2:** Second round of expression screening as fusion proteins.

ORF	M.W. (kDa)	Fusion partner	cloned	IB*	S	M
Rv0011c	10.2	GST	Yes	XX**		
Rv0460	8.1	GST	Yes	XX		
Rv0544c	9.7	GST	Yes	X		
Rv0882	9.6	GST	Yes	X		
Rv1171	15.2	GST	Yes			
Rv2128	7.4	GST	Yes	X		
Rv3078	14.1	GST	Yes			
Rv3632	13.1	GST	Yes			
Rv3656c	7.1	GST	Yes	XXX		
Rv3901c	15.4	GST	Yes	XXX		

Rv0011c	10.2	MBP	Yes	XXX		XXX
Rv0460	8.1	MBP	Yes	XX		XX
Rv0544c	9.7	MBP	Yes	XX		XXX
Rv0882	9.6	MBP	Yes	X		XXX
Rv1171	15.2	MBP	Yes	X		X
Rv2128	7.4	MBP	Yes	XXX		XXX
Rv3078	14.1	MBP	Yes			
Rv3632	13.1	MBP	Yes	X		X
Rv3656c	7.1	MBP	Yes	XXX		
Rv3901c	15.4	MBP	Yes	XX		XXX

Rv0011c	10.2	KSI	Yes	XX		
Rv0460	8.1	KSI	Yes	X		
Rv0544c	9.7	KSI	Yes			
Rv0882	9.6	KSI	Yes			
Rv1171	15.2	KSI	Yes	X		
Rv2128	7.4	KSI	Yes			
Rv3078	14.1	KSI	Yes	X		
Rv3632	13.1	KSI	Yes	X		
Rv3656c	7.1	KSI	Yes	X		
Rv3901c	15.4	KSI	Yes			

* IB: Inclusion body fraction; S: Soluble fraction; M: Membrane fraction.

** Symbol "X" indicates the estimated yield of the fusion protein from Coomassie blue stained SDS-PAGE gel. One "X" corresponds to the expression yield between 10 mg and 20 mg for the fusion protein from 1 L LB culture.

As expected, all of the KSI fusion proteins aggregated in the inclusion body fraction (Table 2, Figure 3). KSI appears to be effective for expression of small peptides that are unstable and/or toxic [29]. The same idea was applied in the present work: KSI forced the unstable or toxic membrane proteins into inclusion bodies, avoiding the proteolysis and/or potential toxic effects to the host cells. Of the ten tested membrane proteins, Rv 3078 was the only one that was exclusively expressed as a KSI fusion protein. Kcv protein, a viral potassium channel, is another example where KSI fusion has been uniquely successful (Qin and Gao, unpublished data).

GST is a highly soluble protein and frequently used as a fusion partner to increase the solubility and the yield of small soluble proteins. GST has been used to fuse with a low molecular weight membrane protein, PsbH [30], and it was found that the majority of the fusion protein was in a soluble state, facilitating the purification by affinity chromatography with immobilized glutathione resin. However, in our experiments, although the molecular weights of the target proteins were less than 16 kDa, all of the expressed fusion proteins were obtained in the inclusion body fraction (Table 2 and Figure 3). It has been previously reported that the capability of GST to increase the solubility of the passenger proteins was weak among the commonly used fusion partners [31,32] consistent with our results. Compared with KSI fusion proteins, GST fusion proteins could be relatively easily solubilized and refolded in detergent micelles, facilitating the cleavage by a specific protease to release the passenger protein prior to further purification [10,22].

MBP is known for its strong capability to increase the solubility of the target protein and has been extensively used as a fusion partner for both soluble and membrane proteins [7,8,26,27]. In our experiments, MBP fusion proteins show two distinguishing features (Table 2 and Figure 3). The MBP fusion proteins were frequently expressed in the membrane fraction, in sharp contrast with the KSI and GST fusion proteins. It is not surprising that MBP increases the solubility of the target protein, but how the MBP fusion tag enhances affinity for the membrane fraction is unclear. In addition, the expression levels of MBP fusion proteins are significantly larger than KSI and GST fusion proteins. Even when the high molecular weight of MBP is taken into account, the expression of the target membrane proteins are still substantially higher than those for KSI and GST fusion proteins. Both features make MBP an attractive fusion partner for membrane proteins: the target membrane proteins are not only overexpressed, but also in the native membrane. Based on these and previous results [7,8], the application of MBP as a fusion partner for membrane proteins is a powerful tool for the study of membrane proteins.

**Figure 4:**
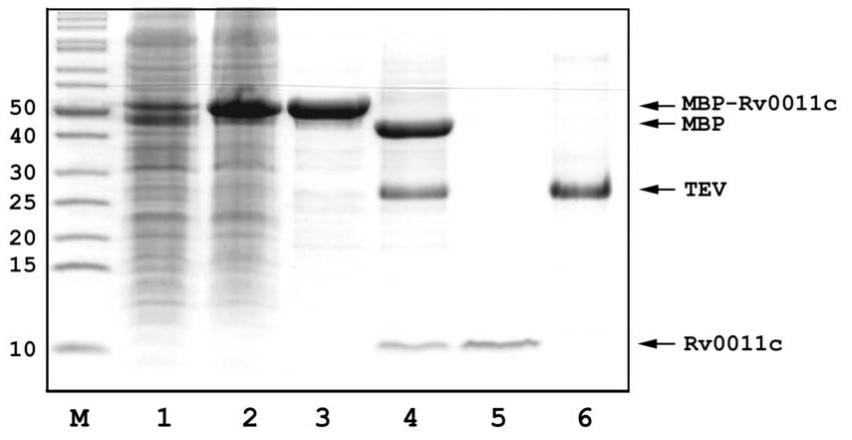
SDS-PAGE analysis of Rv0011c purification. M: protein markers; Lane 1, un-induced whole cells; Lane 2, induced whole cells; Lane 3, purified MBP-Rv0011c; Lane 4, TEV cleavage at 30°C for 2 hours; Lane 5, purified Rv0011c in flow through; Lane 6, purified TEV protease. The molecular weights of the markers are listed to the left (in kDa).

The fusion protein strategy is only effective if the fusion partner can be easily removed. As shown in Figure 4, MBP-Rv0011c was purified to homogeneity and subjected to TEV protease proteolysis. It is clear that after incubation with TEV protease for 2 hours at 30°C, all of the fusion protein was cleaved, resulting in two bands on SDS-PAGE gel corresponding to MBP and Rv0011c, respectively. As previously reported [8,15], removal of MBP and TEV can be simply achieved by passing the sample through a Ni^2+^-NTA column (Figure 4, lane 5). It should be noted that there will be additional three residues (Ser-Asn-Ala) remaining after TEV cleavage at the N terminus of the target protein.

In sum, the second round of expression screening resulted in the production of all the target proteins which were not expressed in the first round of screening. Also unique features of three commonly used fusion partners (KSI, GST and MBP) were illustrated for membrane protein expression. The data shown here strongly suggests that construction optimization, especially with respect to fusion partner protein screening, can be useful for enhancing membrane protein expression.

### A Platform for high throughput cloning and expression screening for membrane proteins

In the present work, the vectors, pTBSG and the three derivative fusion protein expression vectors, are the core components of the platform for high throughput cloning and expression screening for membrane proteins. The platform features the following four advantages:

#### High throughput cloning

Through LIC 95% of the target genes were successfully cloned on the first attempt. The use of identical treatments for all targeted genes makes LIC an ideal method for membrane protein high throughput cloning.

#### High flexibility for different constructs

As shown in the previous section, fusion partners can be very useful for membrane protein expression. Through the use of the same LIC system, the PCR amplified target genes can be inserted into all the vectors for full expression screening, minimizing the efforts associated with a new clone/subclone process. In our structural genomics efforts, both the target genes and vectors were treated and stored as "standard modules", and construct optimization meant combining different modules. In addition, the restriction endonuclease sites for SpeI and KpnI provide an open window for introducing new fusion partners (such as GFP [33]) as new modules, making the present platform updatable.

#### Highly efficient expression

By using the present platform, as shown in our experiments on 41 putative membrane proteins, all of the cloned genes (39) could be expressed and two thirds of the cloned genes (26/39) could be overexpressed in at least one construct. In addition, the target proteins detected only by western blot (not overexpressed) in the first round of screening were not tested as fusion proteins, so there is considerable potential for enhancing the percentage of overexpression.

#### Simplified downstream application

Once cloning and expression screening is completed, downstream purification and characterization can be conveniently performed. In these vectors the N terminal His tag, which is compatible with detergents and chaotic agents, provides a simple and general method for downstream purification, especially for membrane proteins. In addition, for fusion proteins, the fusion partner can be easily removed by passing it through a Ni^2+^-NTA resin after TEV proteolysis, leaving the target protein in the flow through.

## Conclusion

In the present work, a LIC based platform for membrane protein cloning and expression screening is demonstrated on 41 putative integral membrane proteins from the genome of *M. tuberculosis *and potentially suitable for nuclear magnetic resonance characterization. It was found that this platform was characterized by its high throughput and high efficiency for cloning and expression, as well as its high flexibility for construct optimization. It can be anticipated that this platform will be a useful tool for many studies of membrane proteins.

## Abbreviations

GST: glutathione S-transferase; DPC: dodecyl-phosphatidylcholine; IPTG: isopropyl-beta-D-thiogalactopyranoside; KSI: ketosteroid isomerase; LIC: ligation independent cloning; MBP: maltose binding protein; ORFs: open reading frames; TEV: tobacco etch virus protease

## Authors' contributions

HQ carried out experiments and participated in manuscript writing. JH participated in experiments and manuscript writing. YH and SVC participated in the molecular biology experiments. TAC conceived of this study, participated in experimental design and drafted the manuscript. FPG participated in the experimental design and manuscript writing. All authors read and approved the final manuscript.
